# Overexpression of heat shock transcription factor 1 enhances the resistance of melanoma cells to doxorubicin and paclitaxel

**DOI:** 10.1186/1471-2407-13-504

**Published:** 2013-10-29

**Authors:** Natalia Vydra, Agnieszka Toma, Magdalena Glowala-Kosinska, Agnieszka Gogler-Piglowska, Wieslawa Widlak

**Affiliations:** 1 Maria Skłodowska-Curie Memorial Cancer Center and Institute of Oncology, Gliwice Branch, Wybrzeże Armii Krajowej 15, Gliwice, Poland

**Keywords:** Heat shock transcription factor 1, Heat shock proteins, Drug resistance, Doxorubicin

## Abstract

**Background:**

Heat Shock Transcription Factor 1 (HSF1) is activated under stress conditions. In turn, it induces expression of Heat Shock Proteins (HSPs), which are well-known regulators of protein homeostasis. Elevated levels of HSF1 and HSPs were observed in many types of tumors. The aim of the present study was to determine whether HSF1 could have an effect on the survival of cancer cells treated with chemotherapeutic cytotoxic agents.

**Methods:**

We constructed mouse (B16F10) and human (1205Lu, WM793B) melanoma cells overexpressing full or mutant form of human HSF1: a constitutively active one with a deletion in regulatory domain or a dominant negative one with a deletion in the activation domain. The impact of different forms of HSF1 on the expression of *HSP* and *ABC* genes was studied by RT-PCR and Western blotting. Cell cultures were treated with increasing amounts of doxorubicin, paclitaxel, cisplatin, vinblastine or bortezomib. Cell viability was determined by MTT, and IC_50_ was calculated. Cellular accumulation of fluorescent dyes and side population cells were studied using flow cytometry.

**Results:**

Cells overexpressing HSF1 and characterized by increased HSPs accumulation were more resistant to doxorubicin or paclitaxel, but not to cisplatin, vinblastine or bortezomib. This resistance correlated with the enhanced efflux of fluorescent dyes and the increased number of side population cells. The expression of constitutively active mutant HSF1, also resulting in HSPs overproduction, did not reduce the sensitivity of melanoma cells to drugs, unlike in the case of dominant negative form expression. Cells overexpressing a full or dominant negative form of HSF1, but not a constitutively active one, had higher transcription levels of *ABC* genes when compared to control cells.

**Conclusions:**

HSF1 overexpression facilitates the survival of melanoma cells treated with doxorubicin or paclitaxel. However, HSF1-mediated chemoresistance is not dependent on HSPs accumulation but on an increased potential for drug efflux by ABC transporters. Direct transcriptional activity of HSF1 is not necessary for increased expression of *ABC* genes, which is probably mediated by HSF1 regulatory domain.

## Background

HSF1-dependent stress response is an adaptive mechanism which enhances the survival of somatic cells facing diverse arrays of environmental and physiological challenges (such as heat shock, ischemic injury, neurodegeneration, and others) [1,2]. Activation of HSF1 results in induced expression of a set of highly conserved proteins, known as heat shock proteins (HSPs). HSPs act as molecular chaperones by assisting protein folding during their synthesis or repair under proteotoxic conditions. Mammalian HSPs are classified according to molecular size into several families including HSPH (HSP110), HSPC (HSP90), HSPA (HSP70), HSPD (HSP60), and HSPB (small HSPs, sHSPs). Each gene family includes members that are constitutively expressed, inducibly regulated, and/or targeted to different cellular compartments [3].

The primary role of HSF1 in cells is associated with the regulation of *HSPs* expression in response to heat shock or other stress conditions. Moreover, there is some evidence indicating the importance of HSF1 in the processes associated with development, growth and fertility [4-7]. Furthermore, HSF1 facilitates cell survival upon imbalanced cell signaling associated with neoplastic transformation. Convincing evidence of HSF1 involvement in carcinogenesis has emerged from data gathered from a murine tumor model. Namely, lack of HSF1 expression protected mice against tumorigenesis in a chemically-induced skin carcinogenesis model and in a genetic model driven by a clinically relevant oncogenic mutation in p53 (p53R172H) [8]. The role of HSF1 in carcinogenesis includes protecting cancer cells from programmed cell death, overriding cell cycle checkpoints and enhancing metastasis [9-11]. HSF1 also orchestrates a broad network of core cellular functions associated with proliferation, survival, protein synthesis and glucose metabolism, thus enhancing oncogenic transformation [8,9].

Activation of HSF1-dependent stress response, a cytoprotective mechanism, may greatly influence development of an adaptive and protective phenotype in cancer cells subjected to anticancer agents. Elevated expression of HSPs (e.g., HSP90, HSP70, HSP27) has been reported in many types of human malignancies and was linked to cancer resistance to apoptosis induced by chemotherapeutic agents [12-14]. The antiapoptotic function of HSPs was shown for monoblastoid U937 cells and murine fibrosarcoma WEHI-S cells treated with actinomycin-D, camptothecin and etoposide [15] as well as rat brain tumor cells treated with vincristine [16]. In addition, HSP-independent mechanism may be involved in HSF1 regulated resistance of cancer cells to chemotherapeutics. HSF1-binding elements were found in *ABCB1* (*MDR1*) gene promoter coding for P-glycoprotein (P-gp), an energy-dependent drug efflux pump [17,18].

In this study, we established mouse and human melanoma cells overexpressing hHSF1 to study the effect of HSF1 on the survival of cancer cells treated with cytotoxic agents used in chemotherapy. Here, we generated melanoma cells with different mutant forms of human HSF1, leading either to constitutive HSPs activation (transcriptionally active) or lacking the ability to activate HSPs expression (dominant-negative). We also obtained mouse melanoma B16F10 cells with a silenced HSF1 expression. We were thus able to evaluate the contribution of HSF1 and HSPs level in the development of drug resistance by melanoma cells.

## Methods

### Cell lines and cell culture

Melanoma cell lines, B16F10 (mouse), WM793B and 1205Lu (human), were obtained from American Type Cell Culture Collection (ATCC, Manassas, VA). Cells were routinely cultured according to ATCC protocol. Doubling time for B16F10 cells is approximately 24 h, for WM793B and 1205Lu – approximately 48 h. Heat shock was performed by placing plates with logarithmically growing cells in an incubator (Heraeus), at 42°C for 1 hour. For transcriptional studies, cells were allowed to recover at 37°C for 30 minutes or for protein studies were lysed immediately after heat shock or after 6-hour recovery.

### DNA constructs

Human HSF1 (hHSF1) coding sequence (Accession no. NM_005526.2) was amplified by PCR using cDNA from WM793B cells as a template; the sequence recognized by *Hind*III restriction enzyme was introduced into primers. HSF1 cDNA fragment was inserted downstream of the human β-actin promoter into the pHβApr-1-neo expression vector. The hHSF1ΔRD construct containing a constitutively active form of human HSF1 (aHSF1; with 221–315 amino acid deletion) driven by the human β-actin promoter in the pHβApr-1-neo expression vector, was kindly provided by Dr. A. Nakai [6]. A plasmid containing dominant negative human HSF1 (hHSF1-DN; with deletion of amino acids 453–523; [19]) was constructed by PCR-mediated site-directed mutagenesis consisting of two-step PCR, using two overlapping internal primers at the mutagenic site and two outer general primers each flanked by *Hind*III site. The internal primers were as follows: forward 5′-GAGCCCCCCAGGCCTCCCAAGGACCCCACTGTCTTC; reverse 5′-GAAGACAGTGGGGTCCTTGGGAGGCCTGGGGGGCTG. The mutant hHSF1-DN cDNA fragment was inserted downstream of the human β-actin promoter into the pHβApr-1-neo expression vector. The hHSF1, aHSF1, hHSF1-DN sequences were also cloned into the pLNCX2 retrovirus expression vector downstream of the CMV promoter (Clontech). Nucleotide sequence of all constructs was verified by DNA sequencing. Schematic diagram of a structure of analyzed hHSF1 proteins is shown in Additional file 1: Figure S1.

### Stable transfections

Mouse melanoma B16F10 cells were transfected with vectors containing hHSF1, aHSF1, and hHSF1-DN cDNA using Lipofectamine™2000 according to the manufacturer’s protocol (Life Technologies). To select clones that stably express the integrated vector, cells were cultured for 7 days with G-418 (1 mg/ml, Life Technologies). Then, cells were seeded on a 96-well plate (1 cell/well) in the presence of G-418. When colonies were formed, 7–11 individual clones were collected for each construct. Clones expressing the introduced HSF1 (as estimated by Western blotting) were pooled together for further experiments. Stably transfected human melanoma WM793B and 1205Lu cells were obtained by retroviral gene transfer of hHSF1, aHSF1, hHSF1-DN cDNA cloned in the pLNCX2 vector according to the manufacturer’s protocol (Clontech Laboratories, Inc.). Cells were infected in the presence of polybrene (8 μg/ml) and selected in the presence of G-418 (200 μg/ml - WM793B cells, and 400 μg/ml - 1205Lu cells).

### Generation of HSF1-shRNA vectors

The shRNA target sequence for mouse HSF1 was selected using the RNAi Target Sequence Selector (Clontech) and according to a previous report [8]. The target sequences were: HSF1-1 (1856–1876, NM_008296.2) - 5′ GCTGCATACCTGCTGCCTTTA; and HSF1-2 (341–359, NM_008296.2) - 5′AGCACAACAACATGGCTAG. Sense and antisense oligonucleotides were annealed and inserted into the pRNAi-Ready-Siren-RetroQ vector (Clontech) at *Bam*HI/*Eco*RI site. Infectious retroviruses were generated by transfecting DNA into PT67 cells and virus-containing supernatant was collected. Mouse melanoma B16F10 cells were transduced with retroviruses containing HSF1 shRNAs and selected using a medium supplemented with 1 μg/ml puromycin (Life Technologies).

### RNA isolation and RT-PCR

Extraction of total RNA, purification from DNA contamination, synthesis of cDNA and RT-PCR were performed as described in [20]. For RT-PCR 1–2 μl of cDNA template was used and 25–35 cycles were applied depending on the primers set. Quantitative RT-PCR was performed using a Bio-Rad CFX 96TM Real-Time PCR Detection System. A total of 5 pmoles of forward and reverse primers, cDNA template were added to the Real-Time 2× PCR Master Mix SYBR A (A&A Biotechnology, Gdynia, Poland). Primers used in the analyses are listed in Additional file 2: Table S2.

### Protein extraction and Western blotting

Whole cell extracts were prepared using RIPA buffer. Proteins (25 μg) were separated on 8-10% SDS-PAGE gels and blotted to 0.45-μm pore nitrocellulose filter (Millipore) [21]. Primary antibodies against HSF1 (rabbit polyclonal, ADI-SPA-901, Enzo Life Sciences), HSP70 (mouse monoclonal, ADI-SPA-810, Enzo Life Sciences), HSP25 (rabbit polyclonal, ADI-SPA-801, Enzo Life Sciences), HSP105 (rabbit polyclonal, 3390–100, BioVision), or actin (mouse monoclonal, clone C4, MAB1501, Millipore) were used. The primary antibody was detected by appropriate secondary antibody conjugated with horseradish peroxidase (ThermoScientific) and visualized by ECL kit (ThermoScientific).

### Treatment of cells with cytotoxic drugs and MTT assay

Mouse melanoma cells (1.5 × 10^3^/well) or human melanoma cells (4 × 10^3^/well) were seeded in 96-well plates and allowed to attach overnight. Cytotoxic agents: doxorubicin (5, 10, 20, 40, 80 ng/ml), paclitaxel (5, 10, 20, 40, 80 nM), vinblastin (1, 2, 4, 8, 16 nM), cisplatin (2, 4, 8, 16 μM) and bortezomib (2.5, 5, 10, 20 nM) were applied for 48 hours (B16F10 cells) or for 72 h (WM793B and 1205Lu cells). Cell viability was determined by MTT assay, as described in [22]. The absorbance (λ = 570 nm) was read using Synergy 2 microplate reader (Biotek). Relative survival was determined using the formula: viability (%) = (cytotoxic agent treated-blank)/(untreated-blank)*100. All experiments were performed at least in triplicate.

### Assay for the fluorescent dyes efflux

Cells suspended in phenol-free medium supplemented with 0.5% FBS (PAA) in polystyrene tubes were incubated for 30 minutes in a 37°C incubator with (i) doxorubicin (1 μg/ml; 5 *×* 10^5^ cells) or (ii) eFluxx-ID™ Green Detection Reagent (Enzo Life Sciences) (2.5 *×* 10^5^ cells). Next, cells were washed, resuspended in PBS, and analyzed using a FACSCanto cytometer (Becton Dickinson). Dye concentration and treatment exposure times were established experimentally to obtain the best signal-to-noise ratio.

### Side population analysis

Cells were stained according to Goodell’s protocol [23]. Briefly, cells at 1 × 10^6^/ml were suspended in prewarmed phenol-free DMEM (Sigma-Aldrich) with 2% FBS. Hoechst 33342 (Sigma-Aldrich) was added to the final concentration of 5 μg/ml in the presence or absence of verapamil (50 μg/ml; Sigma-Aldrich). Cells were incubated at 37°C for 90 min with intermittent shaking. At the end of incubation, cells were washed with phenol-free DMEM, centrifuged at 4°C, and resuspended in ice-cold PBS. Propidium iodide (Sigma-Aldrich) was added to cells to gate viable cells. Analyses were performed using FACSAria III apparatus (Becton Dickinson). The Hoechst 33342 dye was excited at 357 nm and its fluorescence was dual-wavelength analyzed (blue, 402–446 nm; red, 650–670 nm).

### Statistical analysis

The data were analyzed by Student’s t-test. A p-value of <0.05 was considered statistically significant.

## Results

### Overexpression of HSF1 in melanoma cells results in enhanced survival of cells treated with doxorubicin

**Figure 1:**
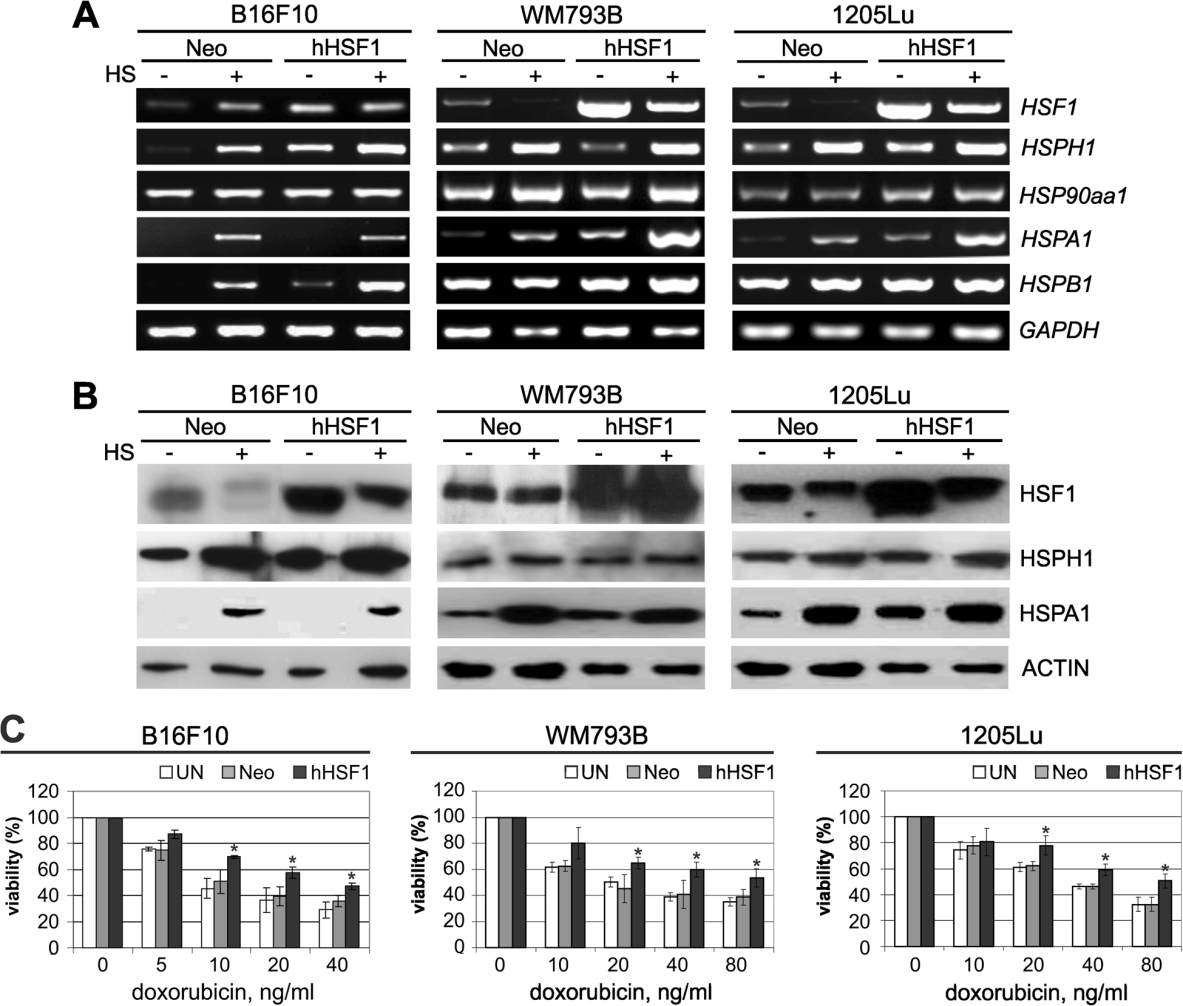
**Overexpression of HSF1 enhances doxorubicin resistance of melanoma cells. A**. Detection of *HSF1* and *HSP* gene transcripts in mouse (B16F10), and human (WM793B and 1205Lu) cells either with stably introduced empty vector (Neo) /control/ or with a vector encoding the full form of human HSF1 (hHSF1). Where indicated, cells were subjected to heat shock (HS) for 1 h at 42°C with subsequent recovery at 37°C for 30 minutes. **B**. Western blot detection of HSF1 and HSPs in cells modified and treated as above. HSF1 was detected directly after HS while HSPs were detected after a 6-hour recovery. **C**. Viability of cells treated with various concentrations of doxorubicin for 48 h (B16F10) or 72 h (WM793B and 1205Lu). Results of the MTT assay are shown in relation to the untreated cells; mean values ± SD from three independent experiments are presented (asterisks indicate p < 0.05).

To determine whether the elevated expression of HSF1 could affect cell response to cytotoxic drugs we established mouse (B16F10) and human (WM793B and 1205Lu) melanoma cells overexpressing the full form of the human HSF1 (hHSF1). Such modified cells were first characterized in the context of HSF1 and HSPs expression at physiological and elevated temperature (heat shock) and compared to cells stably transfected with an empty vector (Neo). We established that HSF1 expression was significantly elevated in hHSF1-overexpressing cells. Moreover, the presence of extra copies of HSF1 in human cells resulted in enhanced expression of inducible HSP70 (HSPA1) already at physiological temperature, and which was visible at both mRNA and protein levels (Figure 1A,B). Although HSPA1 expression was not observed at physiological temperature in mouse B16F10 cells overexpressing hHSF1, the transcription of other HSF1-dependent genes (*Hsph1*, *Hspb1*) was detected in those cells (Figure 1A). Hence, overexpression of the human HSF1 was sufficient to activate some *HSP* genes in mouse and human melanoma cells in heat shock-independent manner.

Next, control and hHSF1-overexpressing mouse and human melanoma cells were treated with increasing concentrations of cytotoxic agents: doxorubicin, vinblastine, paclitaxel, cisplatin or bortezomib for the time period of two cell divisions. Then, cell viability was determined by the MTT assay, and IC_50_ was calculated. Melanoma cells overexpressing hHSF1 showed an enhanced viability after doxorubicin (Figure 1C, Table 1) as well as paclitaxel treatments (Table 1) in comparison to unmodified or Neo control cells. The IC_50_ value for doxorubicin or paclitaxel in cells overexpressing hHSF1 was about 2-fold higher than in control cells. In contrast, the IC_50_ value for cisplatin, vinblastine or bortezomib was not changed due to hHSF1 overexpression (Table 1). We have concluded that overexpression of hHSF1 results in an enhanced viability of cells treated specifically with doxorubicin or paclitaxel.

**Table 1:** **The IC**_**50 **_**values of various chemotherapeutics in mouse and human melanoma cells with different status of HSF1**

	**Doxorubicin,**	**Vinblastine,**	**Paclitaxel,**	**Cisplatin,**	**Bortezomib,**
	**ng/ml**	**nM**	**nM**	**μM**	**nM**
**B16F10**					
Unmodified	10 ± 3,46	4,3 ± 0,71	19 ± 2,64	5,03 ± 1,88	9,73 ± 3,4
Neo	10,8 ± 3,3	3,6 ± 0,87	16 ± 2	6,47 ± 3,41	8,77 ± 1,97
hHSF1	31,5 ± 5,07*	3 ± 0,28	30,67 ± 5,03*	6,77 ± 1,78	10,8 ± 3,68
aHSF1	8,97 ± 1,76	3,13 ± 0,42	20,67 ± 5,03	6,63 ± 2,92	7,63 ± 1,86
hHSF1-DN	39 ± 1,41*	4,7 ± 1,1	29,67 ± 0,58*	6,9 ± 0,14	7,9 ± 1,65
**WM793B**					
Unmodified	20,67 ± 5,77	7 ± 2,65	30,2 ± 5,37	5,6 ± 1,41	3,85 ± 0,07
Neo	17,83 ± 6,33	6,33 ± 1,6	27,47 ± 6,57	4,55 ± 1,34	3,65 ± 0,07
hHSF1	71,33 ± 3,06*	4,75 ± 0,07	58,2 ± 3,11*	5,4 ± 0,71	3,3 ± 0,63
aHSF1	25,33 ± 2,31	7,75 ± 2,47	41,6 ± 6,4	4,3 ± 1,56	3,7 ± 0,14
hHSF1-DN	75 ± 1,41*	5,4 ± 1,41	77 ± 4,24*	7,47 ± 4,1	3,55 ± 0,21
**1205Lu**					
Unmodified	33,47 ± 2,54	2,375 ± 0,04	25 ± 5,55	6,93 ± 1,33	3,2 ± 0,85
Neo	31,13 ± 3,31	2,6 ± 0,96	19,37 ± 7,99	4,37 ± 0,51	3,15 ± 1,06
hHSF1	66 ± 11,31*	3,5 ± 0,14	52,67 ± 7,02*	6,93 ± 2,69	2,55 ± 0,07
aHSF1	24,8 ± 1,7	3,6 ± 1,13	22,33 ± 0,5	5,6 ± 1,64	3,15 ± 0,92
hHSF1-DN	54,5 ± 20,5*	4,67 ± 1,1	38 ± 2*	5,87 ± 1,01	3,65 ± 0,64

Asterisks indicate p < 0.05.

### Efflux of fluorescent dyes is more efficient and side population is increased in cells overexpressing HSF1

**Figure 2:**
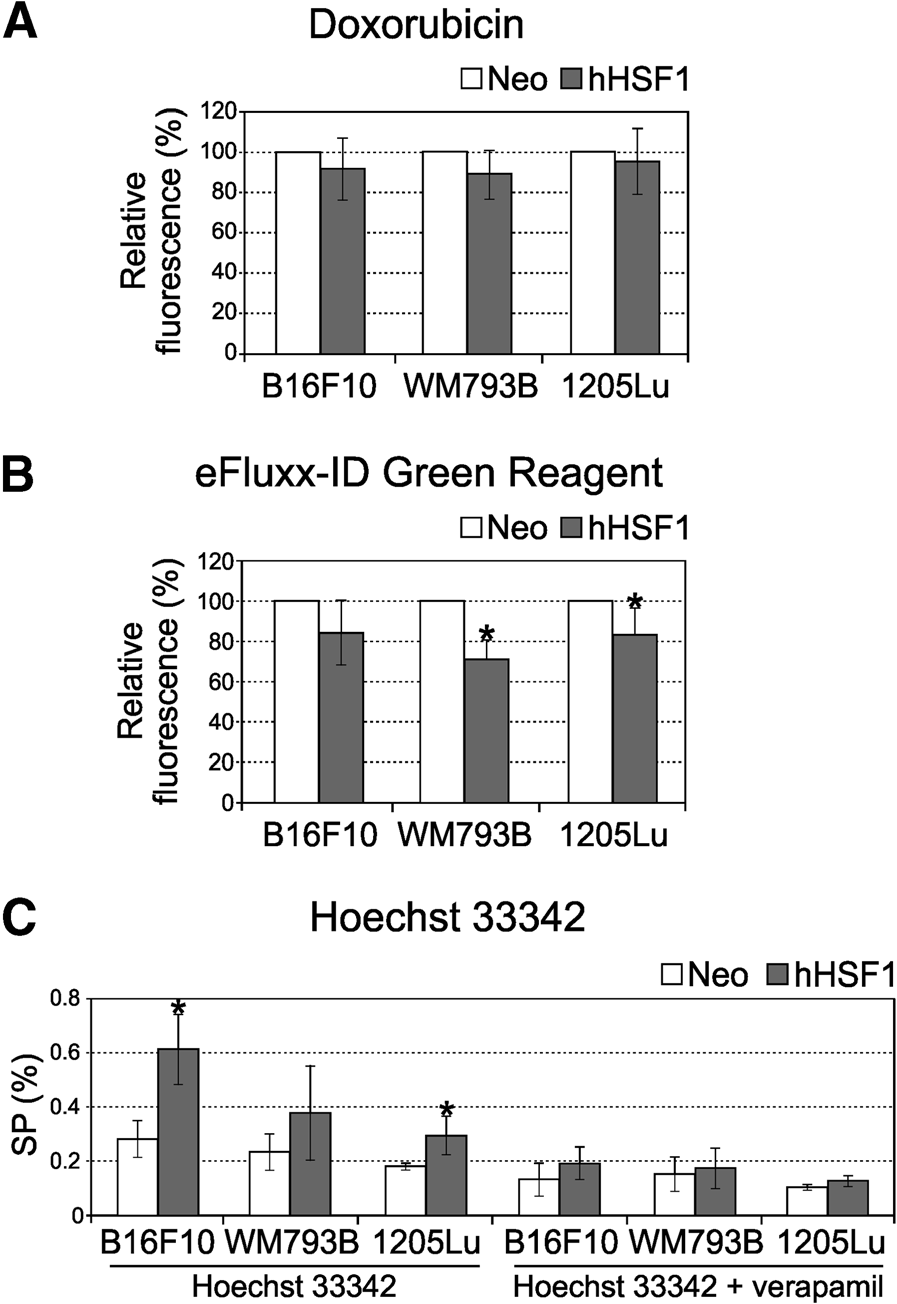
**Fluorescent dyes efflux is enhanced in melanoma cells overexpressing HSF1.** Intracellular fluorescence of doxorubicin **(A)** and eFluxx-ID™ Green Detection Reagent **(B)** in hHSF1-overexpressing cells is shown in relation to control (Neo) cells **(C)**. The percentage of dye-negative cells (side population, SP) following incubation with Hoechst 33342 in the absence or presence of verapamil is presented. Mean values ± SD from at least three experiments are shown (asterisks indicate p < 0.05).

To elucidate mechanisms of acquired doxorubicin resistance of cells overexpressing hHSF1 we estimated accumulation of the drug by flow cytometry. Cells were treated with doxorubicin (1 μg/ml) for 30 minutes and then the doxorubicin fluorescence was checked. Under those conditions doxorubicin accumulation was lower in hHSF1-transduced cells than in control cells, yet observed differences did not reach the level of statistical significance (Figure 2A, see also Additional file 3: Figure S2). The intracellular accumulation of doxorubicin is dependent on the activity of ABCB1 or other proteins belonging to the ABC transporters family. Therefore, we assessed the accumulation of a tracer dye eFluxx-ID™ Green Detection Reagent (Enzo Life Sciences). The reagent is a substrate for three main ABC transporter proteins, ABCB1, ABCC1/ABCC2 and ABCG2 and can serve as an indicator of these proteins’ activity in cells. We found that the dye-specific fluorescence was significantly lower in cells overexpressing hHSF1, and the most effective drug efflux occured in hHSF1-WM793B cells (Figure 2B). This indicates higher activity of ABC transporters in hHSF1-overexpressing melanoma cells leading to more effective drug efflux.

The enhanced ability to efflux of certain dyes such as Hoechst 33342 [23] is a characteristic phenotypic feature of certain kinds of cells, namely side population (SP) cells. We examined the presence of SPs in B16F10, WM793B and 1205Lu cells by staining them with Hoechst 33342 dye in order to generate a Hoechst blue-red profile (see Additional file 4: Figure S3). As a control, verapamil was added which blocks the activity of Hoechst 33342 transporters, and the SP fraction was defined as the dye-free cell fraction diminished in the presence of verapamil. A fraction of SP in control Neo cells ranged from 0.175% to 0.28% of the whole assessed cell population, whereas the number of SP cells was significantly increased in cells overexpressing hHSF1 (>2 fold: an effect especially well noticed in B16F10 line) compared to control cells (Figure 2C). The obtained results suggest that HSF1 overexpression may contribute to the generation of SP phenotype of melanoma cells.

### Expression of constitutively active HSF1 mutant does not enhance resistance to doxorubicin while expression of dominant-negative HSF1 does

**Figure 3:**
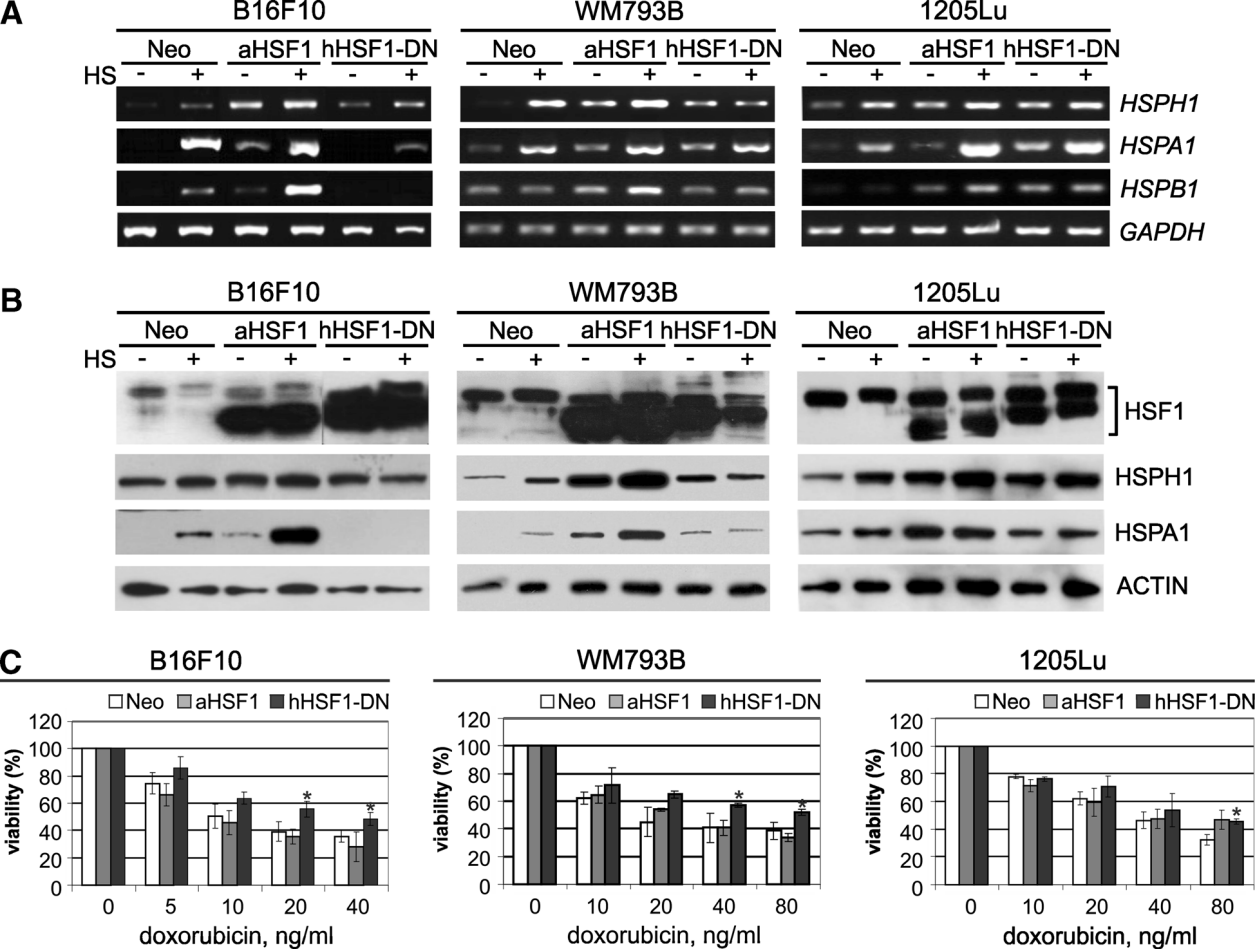
**An increased resistance of melanoma cells to doxorubicin is not coupled with HSF1 transcriptional activity. A**. Detection of transcripts of *HSF1* and *HSP* genes in B16F10, WM793B and 1205Lu cells containing either the empty vector (Neo) or HSF1 mutants. Where indicated, cells were subjected to heat shock (HS) for 1 h at 42°C with subsequent recovery at 37°C for 30 minutes. **B**. Western blot detection of HSF1 and HSPs in cells modified and treated as above. HSF1 was detected directly after HS while HSPs were detected after a 6-hour recovery. **C**. Viability of cells treated with various concentrations of doxorubicin for 48 h (B16F10) or 72 h (WM793B and 1205Lu). Results of the MTT assay are shown in relation to the untreated cells; mean values ± SD from three experiments are presented (asterisks indicate p < 0.05).

To further investigate the mechanism of HSF1-dependent resistance of melanoma cells to doxorubicin we tested two mutant forms of HSF1: constitutively active one and dominant-negative one. The constitutively active form (aHSF1) corresponds to the human HSF1 with a deletion in a heat-responsive regulatory domain (RD; residues 221–315). Dominant-negative form (hHSF1-DN) corresponds to the human HSF1 with a deletion in the C-terminal transcriptional activation domain (residues 453–523) (see Additional file 1: Figure S1). It has been previously shown that deletion of amino acids 221–315 conferred on HSF1 the ability to bind DNA and to induce HSPs expression in the absence of heat shock [6,7], while deletion of amino acids within C-terminal domain led to DNA-binding activity of HSF1 without the ability to activate HSPs expression during heat shock [19]. We established mouse (B16F10) and human (WM793B and 1205Lu) cells overexpressing these mutant forms of HSF1. The shorter mutant forms of HSF1 were present in the modified cells in addition to the longer endogenous HSF1 form (Figure 3). Stably transfected cells were tested for HSPs expression in the absence or after heat shock. Increased expression of several *HSP* genes (*HSPH1*, *HSPB1*, *HSPA1*) was detected in cells overexpressing aHSF1 already at physiological temperature. On the other hand, induction of the same *HSP* genes was partially blocked following hyperthermia in mouse B16F10 cells overexpressing hHSF1-DN (Figure 3A). In human cells, introduction of hHSF1-DN was associated with a slightly higher expression of some HSPs (HSPA1, HSPH1) at physiological temperature, which suggested that introduced dominant negative HSF1 could form heterotrimers with endogenous HSF1 leading to basal transcriptional activity [19,24]. However, in the presence of hHSF1-DN hyperthermia-induced accumulation of HSPs was suppressed in both mouse and human cells (Figure 3B). We have concluded that overexpression of aHSF1 mimicked transcriptional activity of HSF1 during stress conditions, while hHSF1-DN was able to suppress strong induction of HSF1-dependent *HSP* genes normally observed after heat shock, plausibly by blocking the endogenous HSF1 binding.

Cells overexpressing mutant forms of HSF1 were treated with several cytotoxic agents as described previously. Cell viability was determined by the MTT assay and IC_50_ was calculated. We found that overexpression of hHSF1-DN enhanced cell viability following treatment with doxorubicin (Figure 3C, Table 1) or paclitaxel (Table 1), as compared to control. The IC_50_ values for doxorubicin or paclitaxel were around 2-fold higher in cells overexpressing hHSF1-DN and full form of HSF1 (hHSF1) than those observed in control cells, either unmodified or Neo. In contrast, aHSF1-overexpressing cells were unable to confer doxorubicin or paclitaxel resistance, and viability of these cells was the same as that of control cells (Figure 3C, Table 1). The IC_50_ value of cisplatin, vinblastine or bortezomib remained unchanged even though the examined cells overexpressed mutant forms of HSF1 (Table 1). We have concluded that HSF1-associated resistance of melanoma cells treated with doxorubicin or paclitaxel was not coupled to HSPs expression, as cells overexpressing the transcriptionally active form of HSF1 did not acquire resistance to these drugs despite elevated level of HSPs.

### Silencing of HSF1 expression in mouse melanoma B16F10 cells has no significant effect on the survival of cells treated with doxorubicin

**Figure 4:**
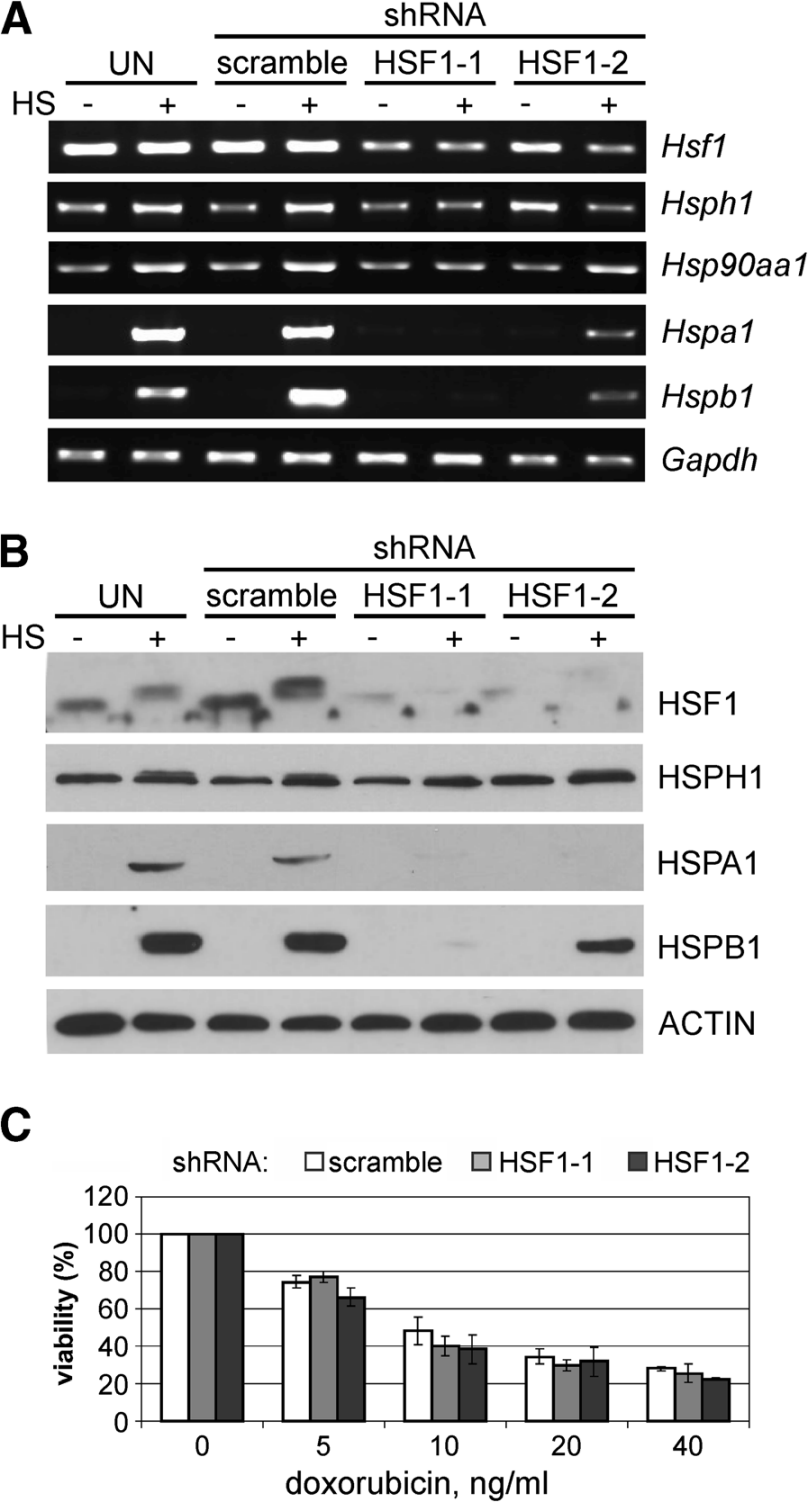
**HSF1 silencing does not influence doxorubicin resistance in mouse melanoma B16F10 cells. A**. Detection of transcripts of *Hsf1* and *Hsp* genes in cells expressing control scrambled shRNA or HSF1-specific shRNAs (HSF1-1, HSF1-2). Where indicated, cells were subjected to heat shock (HS) for 1 h at 42°C with subsequent recovery at 37°C for 30 minutes. **B**. Western blot detection of HSF1 and HSPs in cells expressing control scrambled shRNA or HSF1-specific shRNAs (HSF1-1, HSF1-2). HSF1 was detected directly after HS while HSPs were detected after 6 hours recovery. **C**. Viability of cells treated with various concentrations of doxorubicin for 48 h. Results of the MTT assay are shown in relation to the untreated cells; mean values ± SD from three independent experiments are presented.

We aimed at down-regulating HSF1 expression to determine whether decreased level of HSF1 will reduce the viability of cells following doxorubicin treatment. Two siRNA sequences, complementary to 3′UTR (HSF1-1) or to the coding sequence (HSF1-2) were stably introduced into murine B16F10 cells (stable human cells with silenced HSF1 were not obtained due to lethality). HSF1 and HSPs expression was analyzed by RT-PCR and Western blot in control cells expressing scrambled shRNA and in cells with HSF1-1 and HSF1-2 shRNAs before or after heat shock (Figure 4A,B). Both HSF1-specific shRNA sequences were able to reduce mRNA level and protein level of HSF1. Down regulation of HSF1 expression was connected with a significantly reduced inducibility of *HSP* genes (*Hsph1*, *Hsp90aa1*, *Hspa1* and *Hspb1*) following hyperthermia; of note, shRNA complementary to 3′UTR (HSF1-1) was more effective.

To determine the effect of HSF1 silencing on the sensitivity of B16F10 cells to doxorubicin, cells expressing shRNAs described above were treated for 48 hours with increasing concentrations of doxorubicin (5–40 ng/ml). We observed that cell viability determined using MTT assay was not strongly affected by HSF1 silencing, and was only marginally lower than in the control cells (Figure 4C).

### The mRNA level of several ABC transporters is increased in cells overexpressing HSF1 and its dominant negative form

**Figure 5:**
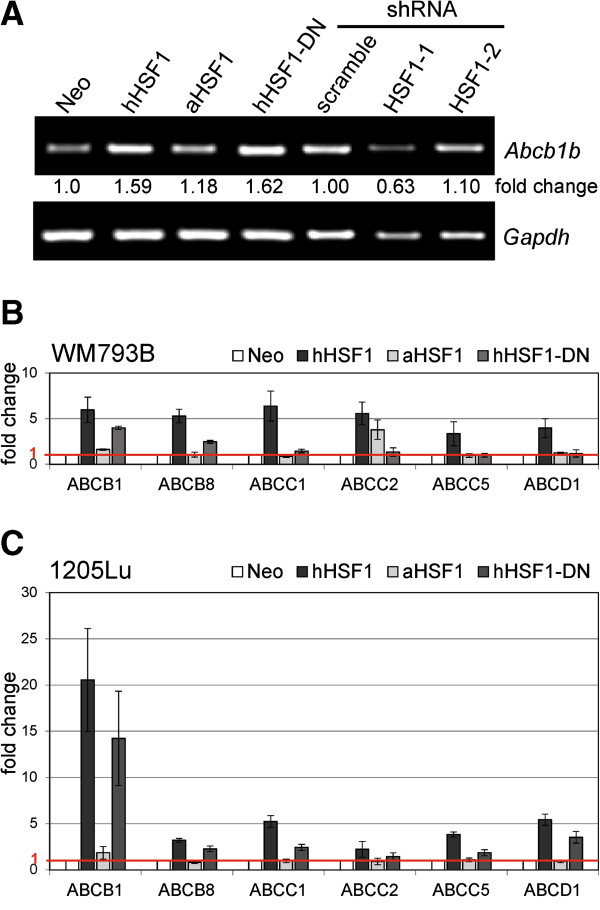
**Expression of several ABC transporters is increased in cells overexpressing full HSF1 or its dominant negative form.** Changes in *ABC* transporter genes expression were estimated based on semi-quantitative RT-PCR (after gel densitometry) in B16F10 cells **(A)** or using quantitative RT-PCR in WM793B **(B)** and 1205Lu **(C)** human cells. Fold changes were calculated in relation to expression levels in control (Neo) cells (1.0 value represented by a horizontal red line) after normalization against *GAPDH* gene expression. Results represent mean values ± SD from three experiments.

Increased efflux of drugs mediated by the ABC transporters is the most commonly encountered mechanism of drug resistance. We analyzed the expression of several ABC transporters in melanoma cells having different HSF1 status. We selected ABCB1, ABCC1, ABCC2, ABCC5, ABCB8, ABCD1 transporters, which were previously reported to be involved in doxorubicin resistance [25,26]. In cells overexpressing hHSF1 the most prominent was up-regulation of *Abcb1b/ABCB1* gene transcription observed in both mouse and human cells (Figure 5). Transcription of other analyzed *ABC* genes (namely *ABCB8*, *ABCC1*, *ABCC2*, *ABCC5* and *ABCD1*) was significantly elevated in human cells overexpressing hHSF1 (Figure 5B,C), but not in mouse cells (data not shown). We could not confirm differences in ABC protein levels between control and hHSF1-overexpressing cells due to unsatisfactory specificities of available antibodies, which showed substantial cross-reactivity to other proteins.

The level of *Abcb1b*/*ABCB1* mRNA in cells overexpressing hHSF1-DN, both mouse and human, was also higher than in control Neo cells and cells overexpressing aHSF1 form (Figure 5). Transcripts of some other ABC transporters were more abundant in human cells overexpressing hHSF1-DN compared to control cells (Figure 5B,C). When the level of *Abcb1b* gene transcript was tested in B16F10 cells with silenced HSF1 we found its reduced expression only in the case of shRNA complementary to 3′UTR (HSF1-1) (Figure 5A). We concluded that expression of *ABC* genes was significantly increased in cells overexpressing hHSF1-DN form despite lack of direct transcriptional effect on *HSP* genes. Importantly, cells overexpressing hHSF1-DN showed an enhanced potential for drug efflux (tested with eFluxx-ID^TM^ Green Detection Reagent; see Additional file 5: Figure S4). The obtained results suggest importance of HSF1 regulatory domain (absent in aHSF1 form) for enhanced *ABC* expression and drug resistance.

## Discussion

High levels of HSF1 and HSPs expression were observed in a broad range of human tumors [27-30]. Moreover, it has been shown that increased HSF1 expression is associated with reduced survival of cancer patients. It is not surprising as HSF1 modulates an entire network of cellular functions that enable neoplastic transformation [8,31]. However, the impact of HSF1 overexpression on cell susceptibility to chemotherapy has not been studied so far. Chemotherapy, a major modality of cancer treatment, is effective initially in controlling the growth of many sensitive tumors, but later it often fails due to the development of resistance to the received drugs. Diverse mechanisms are involved in the acquisition of drug resistance by cancer cells. Understanding them is the key to identify new possible treatments.

In the presented work, we screened the sensitivity of mouse (B16F10) and human (WM793B and 1205Lu) melanoma cells overexpressing HSF1 to different anticancer drugs. We found that HSF1 overexpression had no effect on the survival of cells treated with cisplatin, vinblastine or bortezomib, while the survival of cells treated with doxorubicin or paclitaxel was significantly enhanced when compared to their parental wild-type cells (or control cells containing the empty vector). Surprisingly, we revealed that such selective resistance of melanoma cells was not dependent on direct transcriptional activity of HSF1 (and linked HSPs expression and accumulation). Melanoma cells expressing transcriptionally competent and constitutively active HSF1 mutant characterized by an enhanced expression of HSPs did not acquire resistance. On the other hand, HSF1 mutant form with a deletion in the transcriptional activation domain was found to be as effective as overexpression of wild-type HSF1.

The primary role of HSF1 is traditionally referred to the regulation of *HSP* genes expression. It is generally accepted that HSPs are the fundamental component of cytoprotective reaction that enables somatic cells to survive exposure to harmful conditions. HSPs prevent protein denaturation and/or processing of denatured proteins, which limits accumulation of misfolded species [32,33]. Other mechanism of HSP-dependent cytoprotection involves inhibition of apoptosis. Direct physical interactions with apoptotic molecules were demonstrated for HSPA1, HSPB1 and HSP90 [34,35]. Regardless of well-known cytoprotective function of HSPs, its role in the effectiveness of chemotherapy is not obvious. There are several reports showing that up-regulation of HSP90, HSPA1 or HSPB1 is associated with cell resistance to cisplatin or doxorubicin [36-39]. Furthermore, the damage induced by doxorubicin is more efficiently repaired following heat shock, which correlates with nuclear translocation of HSPB1 and HSPA1 [40]. Also, it was reported that heat-induced carboplatin resistance of p53-dependent hepatoma cells is mediated by HSPA1 [41]. Nevertheless, there are several reports demonstrating that activation of HSPs expression does not enhance cancer cell survival in various types of neoplasia upon cisplatin, colchicine, 5-fluorouracil, actinomycin D or methotrexate treatments [42-46]. Moreover, diminished HSPs expression resulting from HSF1 silencing did not abrogate resistance of cervix carcinoma HeLa cells to cisplatin [47]. Thus, it seems plausible that susceptibility of cells to chemotherapeutics does not solely depend on HSPs expression. The presence of HSPs could be just a secondary effect of HSF1 activity, while mechanisms of HSF1-dependent resistance of cancer cells to drugs could be connected to its interactions with other proteins and/or its impact (direct or indirect) on expression of non-*HSPs* genes. If fact, it was already reported that HSF1 interacts with p53 and enhances p53-mediated transcription [48] or regulates expression of ATG7 (autophagy-related protein 7) [49]. Recent studies have shown that although HSPs expression is important for the tumor initiation [50], a network of genes regulated by HSF1 in malignant cells is distinct from the transcriptional program induced by heat shock [51].

In this report we show that enhanced resistance to doxorubicin and paclitaxel is associated with enhanced drug efflux. Most markedly, the ABC transporter substrate eFluxx-ID Green Reagent was more effectively removed from cells overexpressing HSF1. We found that transcription of several ABC transporters was increased not only in cells overexpressing HSF1 but also its dominant negative form, while not the constitutively active form. This finding suggests that enhanced expression of *ABC* genes is not coupled directly to transcriptional activity of HSF1. The expression of *Abcb1b*/*ABCB1* gene was mostly dependent on HSF1 in all three tested melanoma cell lines. It has been previously demonstrated that multidrug resistance of osteosarcoma U2-OS cells and hepatoma HepG2 cells was mediated by HSF1-dependent expression of the *ABCB1* gene, but not by HSPs expression [52]. Additionally, the transcriptional activity of HSF1 has been required for enhanced expression of *ABCB1* gene in HeLa cells [18]. Although HSE (heat shock element) sequences are present in *ABCB1* gene promoter [17], it was revealed that the mere binding of HSF1 was not sufficient to transactivate the *ABCB1* expression, as it was in the case of *HSP* genes [18,19]. Hence, a plausible posttranscriptional mechanism of *ABCB1* up-regulation in HSF1 overexpressing cells has been proposed [52].

Different mechanisms explaining HSF1 influence on *ABC* mRNAs up-regulation may be proposed. Our data indicate that the HSF1 regulatory domain, which confers repression at control temperature and heat inducibility of HSF1 is required for this effect. It could be hypothesized that HSF1 mediates, *via* its regulatory domain, the activity of other transcription factors or that it affects mRNA maturation or stability. Although a role for HSF1 in RNA processing has not been fully documented, HSF1 incorporation into nuclear stress bodies, where RNA splicing could take place, was reported [53]. Recently, it was shown that HSF1 is involved in the regulation of mRNA-binding protein ELAVL1 (HuR) which, in turn, controls mRNA stability and/or translation of many proteins involved in cancer [54]. In spite of HSF1-dependent accumulation of *Abc*/*ABC* transcripts we did not confirm the corresponding accumulation of ABC proteins. However, our data confirm an enhanced drug efflux, which is considered to be the most relevant indicator of both expression of ABC transporters and its molecular catalytic activity [55,56].

## Conclusions

The results of our study indicate that melanoma cells with HSF1 overexpression are more resistant to doxorubicin or paclitaxel. Such HSF1-mediated drug resistance is not dependent on HSPs accumulation but is rather associated with increased drug efflux mediated by ABC transporters. However, direct transcriptional activity of HSF1 is not necessary for increased *ABC* genes expression. We assume that HSF1, but not HSF1-induced HSPs expression, is critical for the observed selectively enhanced drug resistance.

## Abbreviations

HSF1: Heat shock transcription factor 1; HSP: Heat shock protein; ABC transporter: ATP-binding cassette transporter; ATG7: Autophagy related protein 7.

## Competing interests

The authors declare that they have no competing interests.

## Authors’ contributions

NV carried out most of the molecular biology experiments, designed the study and drafted the manuscript. AT participated in the construction and characteristization of cell lines. MG-K performed analysis of fluorescent dye accumulation by flow cytometry. AG-P participated in the analysis of ABC transporters’ expression. WW designed and wrote the manuscript. All authors read and approved the final manuscript.

## Pre-publication history

The pre-publication history for this paper can be accessed here:

http://www.biomedcentral.com/1471-2407/13/504/prepub

## Supplementary Material

Additional file 1: Figure S1 Structure of wild-type human HSF1 protein and the corresponding mutants: constitutively active form (aHSF1) and dominant negative form (hHFS1-DN). DBD – DNA-binding domain, HR-A/B, HR-C – hydrophobic repeats, AD – C-terminal transcription activation domain. Numbering refers to the amino acids at the borders of the domains. Click here for file

Additional file 2: Table S1 Characteristics of primers used in RT-PCR analyses. Click here for file

Additional file 3: Figure S2 Representative histograms from flow cytometric analysis of cellular accumulation of doxorubicin **(A)** and eFluxx-ID™ Green Detection Reagent **(B)** in control (Neo) and hHSF1-expressing cells. Click here for file

Additional file 4: Figure S3 Representative FACS dot plot showing the presence and phenotype of SP cells in melanoma cells expressing the empty vector (Neo) and hHSF1 (hHSF1). Cells were stained with Hoechst 33342 in the absence **(A)** or presence **(B)** of verapamil. Small gated cell population identifies the SP **(A)** that disappear in the presence of verapamil **(B)**. Click here for file

Additional file 5: Figure S4 Intracellular fluorescence of eFluxx-ID™ Green Detection Reagent in cells with different status of HSF1 in relation to control (Neo) cells. Mean values ± SD from at least three experiments are shown (asterisks indicate p < 0.05). Click here for file
