# Supplementary figures and images for: Overexpression of heat shock transcription factor 1 enhances the resistance of melanoma cells to doxorubicin and paclitaxel
Source: BMC Cancer. 2013 Oct 29;13:504. doi: 10.1186/1471-2407-13-504 (PMC4231344; doi:10.1186/1471-2407-13-504)

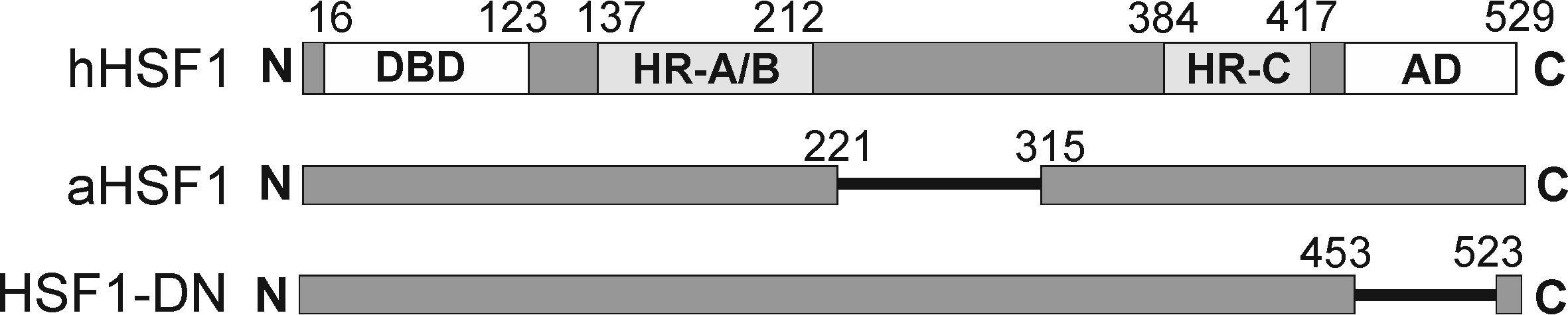

Supplement: Additional file 1: Figure S1 — Structure of wild-type human HSF1 protein and the corresponding mutants: constitutively active form (aHSF1) and dominant negative form (hHFS1-DN). DBD – DNA-binding domain, HR-A/B, HR-C – hydrophobic repeats, AD – C-terminal transcription activation domain. Numbering refers to the amino acids at the borders of the domains. [file 1471-2407-13-504-S1.tiff]

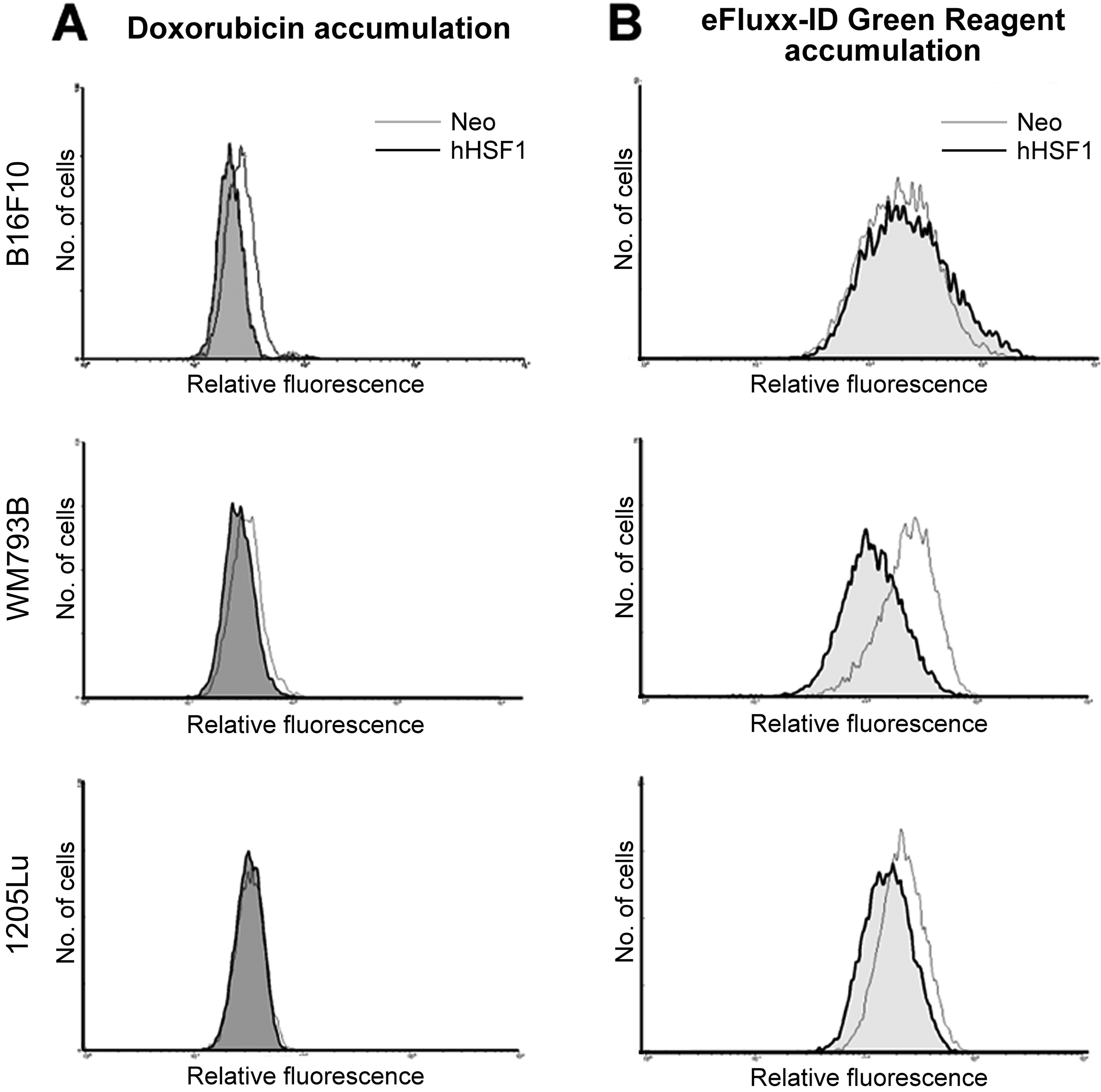

Supplement: Additional file 3: Figure S2 — Representative histograms from flow cytometric analysis of cellular accumulation of doxorubicin (A) and eFluxx-ID™ Green Detection Reagent (B) in control (Neo) and hHSF1-expressing cells. [file 1471-2407-13-504-S3.tiff]

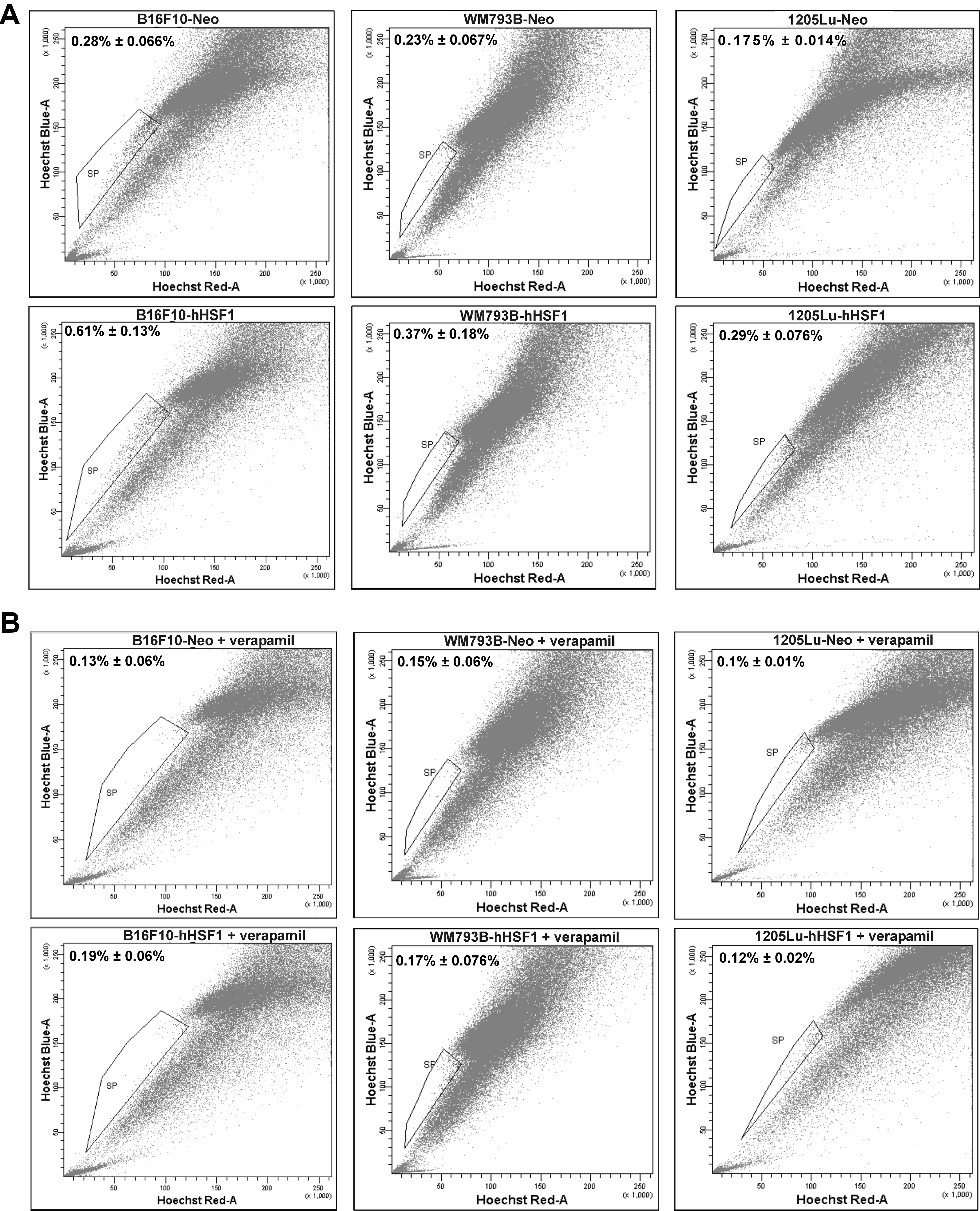

Supplement: Additional file 4: Figure S3 — Representative FACS dot plot showing the presence and phenotype of SP cells in melanoma cells expressing the empty vector (Neo) and hHSF1 (hHSF1). Cells were stained with Hoechst 33342 in the absence (A) or presence (B) of verapamil. Small gated cell population identifies the SP (A) that disappear in the presence of verapamil (B). [file 1471-2407-13-504-S4.tiff]
