# Supplementary material for: Overexpression of heat shock transcription factor 1 enhances the resistance of melanoma cells to doxorubicin and paclitaxel
Source: BMC Cancer. 2013 Oct 29;13:504. doi: 10.1186/1471-2407-13-504 (PMC4231344; doi:10.1186/1471-2407-13-504)
Supplement: Additional file 2: Table S1 — Characteristics of primers used in RT-PCR analyses. [file 1471-2407-13-504-S2.doc]

**Additional file1: Table S1.** . Characteristics of primers used in RT-PCR analyses.

| **A species** | **Gene Symbol** | **GeneBank**  **Acc.No.** | **Primer sequence**  **(5’ → 3’)** | **Location of amplified region** | **Product size**  **(bp)** |
| --- | --- | --- | --- | --- | --- |
| mouse | *Hsf1* | NM_008296 | F: tgtccccaactgccttcatt  R: gtccatggcatccaggtgat | 1130 – 1320 | 190 |
|  | *Hspa1a*  *Hspa1b* | NM_010479.2  NM_010478.2 | F: ccatccagagacaagcgaag  R: cgtttagaccgccgatcaca | 17 – 736 | 719 |
|  | *Hspb1* | NM_013560 | F: cctcttcgatcaagctttcg  R: gccttccttggtcttcactg | 224 – 500 | 276 |
|  | *Hsp90aa1* | NM_010480 | F: ggcatgaaaactaaggggaag  R: agggttgttctcgggacttt | 2443 – 2754 | 311 |
|  | *Hsph1* | NM_013559 | F: agttgcagccaaaaaccagca  R: ccagcacagaccttcgctc | 386 – 711 | 325 |
|  | *Abcb1b* | NM_011075 | F: tcccagagtgacactgatgc  R: cagccccataaccagaaaga | 2076 – 2435 | 359 |
|  | *Abcc1* | NM_008576 | F: gggctttgtgtcagctttgc  R: ccggatggtggactggataa | 4256- 4448 | 192 |
|  | *Abcc2* | NM_013806 | F: ccgtgcttcgaaaatccaaa  R: gggacccatattggacagca | 4439 – 4671 | 232 |
|  | *Abcc5* | NM_013790 | F: ccttcaccatcaagcccaag  R: gtgccactgaacagcactgg | 3837 – 4044 | 207 |
|  | *Abcd1* | NM_007435 | F: gctgcagtttgatggggaag  R: caggggttgaactggagctg | 2454 – 2634 | 180 |
|  | *Abcb8* | NM_029020 | F: ggggccaggtcataggtttc  R: tctggccaccagacaaggtt | 1926 – 2135 | 209 |
|  | *Gapdh* | NM_008084 | F: tggtgaagcaggcatctgagg  R: catgaggtccaccacccttgt | 826 – 1028 | 202 |
| human | *HSF1* | NM_005526 | F: ccagcaacagaaagtcgtca  R: gagctcattcttgtccaggc | 710 – 1322 | 612 |
|  | *HSPA1A,*  *HSPA1B* | NM_005345,  NM_005346.4 | F: cgccgtttccagcccccagtc  R: cgttgagccccgcgatcaca | 191 – 748  164 – 721 | 557 |
|  | *HSPB1* | NM_001540 | F: agagcagagtcagccagcat  R: gttgacatccagggacacg | 138 – 461 | 323 |
|  | *HSP90aa1* | NM_001017963 | F: cagcctttgtggaacgtctt  R: ctctccatgtttgctgtcca | 2225 – 2546 | 321 |
|  | *HSPH1* | NM_006644 | F: aagttgaccagcctccagaa  R: tggtccacacagcttgtctc | 2128 – 2354 | 226 |
|  | *ABCB1* | NM_000927 | F: ccatggccgtggggcaagtc  R: agccagcgtctggccttct | 3447 – 3694 | 247 |
|  | *ABCC1* | NM_004996 | F: gagaaaaggtcggcatcgtg  R: ggagggaacccgaaaacaaa | 4134 – 4328 | 194 |
|  | *ABCC2* | NM_000392 | F: tcattcagacgaccatccaa  R: ttctcaatgccagcttcctt | 4560 – 4755 | 195 |
|  | *ABCC5* | NM_005688 | F: cagctgccatggacacagag  R: aatcgggaactgtcgttgga | 4235 – 4430 | 195 |
|  | *ABCD1* | NM_000033 | F: tacccggactcagtggagga  R: catccaggagggcgtactttg | 2074 – 2287 | 213 |
|  | *ABCB8* | NM_007188 | F: cacggtgctgatactggatg  R: tatagcccgcctttcttcag | 1932 – 2150 | 218 |
|  | *GAPDH* | NM_002046 | F: cgtcttcaccaccatggaga  R: cggccatcacgccacagttt | 498 – 773 | 275 |
